# Evaluation of airborne total suspended particulates and heavy metals in anodizing and electroplating surface treatment process

**DOI:** 10.1038/s41598-021-01577-9

**Published:** 2021-11-18

**Authors:** Donghyeon Kim, Sungyo Jung, Chungsik Yoon

**Affiliations:** 1 Institute of Health and Environment, Seoul National University, Seoul, South Korea; 2 Department of Environmental Health Sciences, Graduate School of Public Health, Seoul National University, Seoul, South Korea

**Keywords:** Health occupations, Risk factors

## Abstract

This study is to evaluate exposure to harmful substances, such as particulate and heavy metals, by considering various factors, in anodizing and electroplating surface treatment process. Exposure evaluation studies on seven heavy metals (Cr, Zn, Ni, Pb, Cd, Al, and Ba) and total suspended particulates (TSP) were conducted. Heavy metals were analyzed using inductively coupled plasma mass spectrometry (ICP-MS). This study also checked the ventilation volume of the hood with a thermal anemometer. Measurement was conducted for 8 h and 8 days. The sample number, N, of the heavy metals and TSP was 107. Geometric mean (GM) of TSP during Cr plating process was 6.15(GSD, 3.35) mg/m^3^. GM of Cr during Cr plating was 1.86(GSD, 6.65) mg/m^3^. GM of TSP and heavy metals differences were statistically significant for each process and date variation. Average ventilation volume for all hoods ranged from 1.20 to 4.98 m^3^/s. In the hood 30 cm from bath, ventilation was 0.1 times lower. Increasing ventilation volume of the hood was the most influential factor, followed by machine operation time and workload. The high concentration was due to low ventilation suction flow. We can improve health to reduce exposure by resolving the fundamental cause of risk occurrence.

## Introduction

Among the metal material industry, the surface treatment industry improves the product quality of protective surfaces by using electrical, physical, and chemical treatment methods on two surface materials to prevent rust, beautify appearance, and increase wear resistance, electrical insulation, and electrical conductivity^1^.

As anodizing, the cathode is made of Al alloy, Pb, or stainless steel. Anodizing is applied to improve corrosion by an anode + oxidizing, thereby undergoing degreasing, neutralization as a pre-treatment process, coloring, sealing, polishing, and non-polishing as a post-treatment process. The oxide film is formed by soft anodizing and hard anodizing. As a post-treatment process, the product is completed through neutralization, water cleaning, drying, and packaging^2^.

The most important exposed materials in the anodizing process are heavy metals. During the process of oxide film formation, abundant Al depending on the type of Al alloy are used, and exposure to other heavy metals is possible. The artificial exposure route of Al is through the respiratory tract, Al is related to potroom asthma, chronic bronchitis, pulmonary fibroids, and granulomatous lung diseases upon exposure through inhalation^3^. Al exposure was significantly correlated with various neurological disorders, and contact dermatitis in workers exposed to Al alloy and Al dust also has been reported. Al toxicity can occur as a result of the interaction between Al and the plasma membrane with target established by the body^4^. Al inhaled through the respiratory tract is replaced by magnesium and iron, resulting in intercellular exchange, cell growth, and secretion functions. The changes in neurons by Al are similar to the degenerative lesions observed in Alzheimer's patients, and complication of Al toxicity may have neurotoxic effects, such as nerve atrophy of the cerebellum, black matter, and striatum^5^. Similarly, high concentrations of Al are very toxic to aquatic animals, especially gill-respiring organisms such as fish, and can destroy plasma and bloodstream ions, and cause osmotic disorders^6^.

Chromium plating was used by an electrolytic solution of chromic acid during the plating process. Workers can be exposed to Hexavalent chromium (Cr (VI)) during the mixing of acid powders and carrying products^7^.

Hexavalent chromium (Cr (VI)) compounds are occupational carcinogens that cause lung, nasal, and sinus cancers^8–10^. Cr (VI) compounds are produced from other airborne forms of Cr industries that use Cr (VI) compounds, such as steel passivation, electroplating, stainless steel welding, and paints production, Cr-based pigments, fungicides, and anti-corrosion compounds as a by-product^11^. Exposure to Cr during electroplating is a characteristic cause of occupational asthma. Sensitivity to Cr in electroplates may occur in situations where exposure levels are likely to be within the current exposure standards^12^.

Previous similar studies on plating show that the metal surface is plated by shooting electrons from the cathode (Eq. 1;^13^). For anodizing, the surface treatment principle is different; the surface of the metals is anodized by shooting oxygen from the anode^14^. The reactions occur simultaneously during anodizing^3^ (Eqs. 2, 3).

-plating equation.

1 $$ {\text{M}}^{{{\text{n}} + }} + {\text{ne}}^{ - } \to {\text{ M}}^{{\text{o}}} $$

where M^n+^ is ion, n is number of moles reacting, and e^-^ is electron and M^o^ is the metals.

-anodizing equation (oxidation and dissolution).

2 $$ {\text{2Al}} + {\text{3H}}_{{2}} {\text{O}} \to {\text{Al}}_{{2}} {\text{O}}_{{3}} + {\text{6H}}^{ + } + {\text{6e}}^{ - } $$

3 $$ {\text{Al}}_{{2}} {\text{O}}_{{3}} + {\text{6H}}^{ + } \to {\text{ 2Al}}^{{{3} + }} + {\text{3H}}_{{2}} {\text{O}} $$

This study evaluated the exposure of each process of business sites performing Al anodizing and Cr electroplating. Exposure evaluation studies on seven heavy metals (Cr, Zn, Ni, Pb, Cd, Al, and Ba) and TSP were conducted. This study also evaluated harmful substances by checking the ventilation volume of the hoods. The sample number, N, for TSP and heavy metals is 107.

## Materials and methods

Table 1 shows the temperature, operations time, and electrolyte chemical composition of the bath in all the workplace processes. There are three standard surface treatment baths at this workplace which were used; the number 1 represents the medium-sized bath, and the number 2 represents the large-sized bath. Table S1 lists the values ​​for special processes that require specific pH or voltage adjustments and provides information on temperature and humidity during the working environment measurement period. Table S2 shows the amount of work and the number of workers during the work environment measurement period, which can be important factors for identifying variable factors according to concentration.

**Table 1:** Temperature, operation time and used chemical in all the processes.

	Temperature (℃)	Operation (sec)^a^	Electrolyte composition (chemical)
Degreasing	45–55	30–90	Na_2_CO_3_, Na_3_PO_4_, NaC_2_HCl_3_, C_2_Cl_4_
Etching & Neutralization	55–65	30–60	NaOH, H_2_SO_4_, HF, HNO_3_
Soft anodizing_1*	21–27	2,400–2,700	H_2_SO_4_, Al_2_O_3_
Soft anodizing_2**	21–27	2,400–2,700	H_2_SO_4_, Al_2_O_3_
Hard anodizing_1*	− 4–4	312–432	H_2_SO_4_, Al_2_O_3_
Hard anodizing_2**	− 4–4	312–432	H_2_SO_4_, Al_2_O_3_
Chromium plating	65–75	600–720	H_2_SO_4_, H_2_CrO_4_
Coloring 1*	40–60	600–720	Azo
Coloring 2**	40–60	600–720	Azo
Sealing	65–75	420–780	C_2_H_3_NaO_2_ (12%), Ni(CH_3_CO_2_)_2_ (5%)
Polishing	100–120	60–180	H_2_SO_4_, HNO_3_, Al_2_O_3_, H_2_CrO_4_
Non-polishing	45–55	60–90	H_2_SO_4_, HNO_3_, Al_2_O_3_, H_2_CrO_4_
Chromium cleaning	21–27	180–300	CH_2_Cl_2_

*1 = Medium size of bath.

**2 = Large size of bath.

^a^Time(second) to make 1 product.

**Figure 1:**
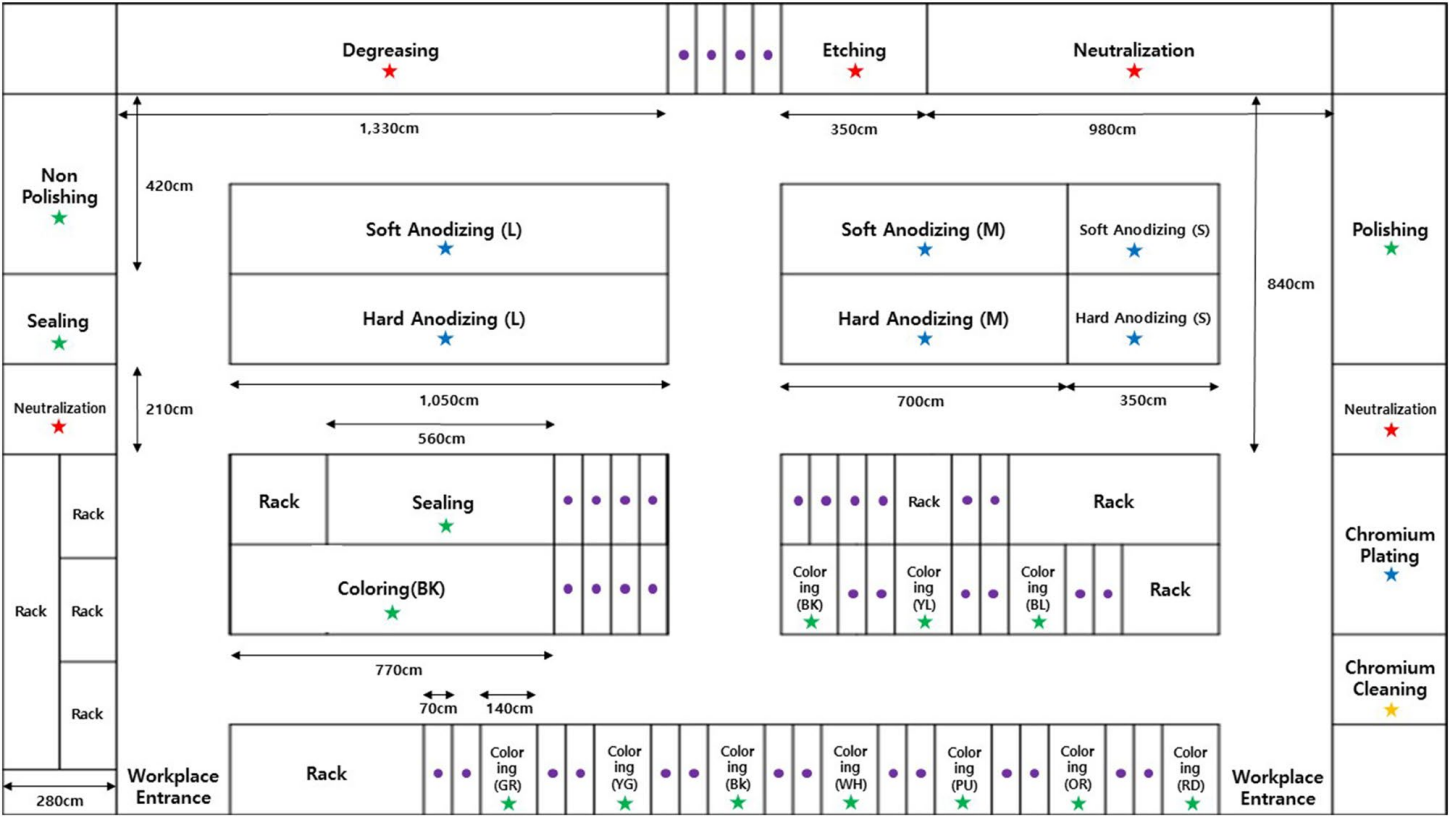
Anodizing and Electroplating schematic and sample location 1: the icons are the sampling locations (red star: pre-treatment process, blue star: anodizing and electroplating process, green star: post-treatment process, orange star: other processes, purple circle: cleaning process). *RD* Red, *OR* Orange, *YL* Yellow, *GR* Green, *BL* Blue, *PU* Purple, *YG* Yellow Green, *WH* White. Workplace (35 m × 17.85 m).

The workplace in this study addressed all processes for anodizing and Cr plating, and the processes can be largely divided into pre-treatment, anodizing, Cr plating, and post-treatment processes. There are three types of surface treatment baths: small, medium and large. But small baths are rarely used in the process. So, to reflect realistic exposure, medium and large baths were evaluated except for small baths (Fig. 1). Drying, packaging, and assembling processes are connected to the above processes (Figure S1).

Samples were collected at a height of 1.5 m to represent the breathing zone of workers. The measurement time proceeded from 8:30 am to 5:30 pm for 8 days, and the measurement was conducted for total of 8 h, excluding breaktime. As a control, the anodizing and plating control sample and drying and packaging control sample were placed in the workplace to check the blank.

### Air sampling and analysis of TSP

PVC (Polyvinyl Chloride) filter (37 mm, pore size 5um, SKC, USA) in the three-piece 37 mm closed face plastic cassette (Whatman Grade QM-A, 37 mm; Whatman, Maidstone, UK) was used to measure fine dust generated during the operation of forming an oxide film and plating, which were the main processes during anodizing and electroplating. These were connected by two types of air sampling pump (Casella APEX-2, USA & Gillian, USA) operating at an airflow rate of 2 ℓ / min. The sample was wrapped with Parafilm (Bemis Company, USA) before and after the sample was collected so as not to be exposed to outside air and then stored to room temperature.

To analyze the TSP, PVC filter was contained in a desiccator for more than 24 h, and then weighed using an analytical balance having 0.01 μg sensitivity (Mettler XP6 Microbalance, Mettler Toledo, Hightstown NJ, USA). Before measuring weight of the filter, static electricity was removed and weight change according to daily temperature and humidity change was corrected using a blank filter. Weight was measured 3 times per sample, and the average value was used as the final weight concentration. High flow rate pump was calibrated before and after sampling using a flow calibrator (Drycal, Defender 520-M, MesaLabs, USA).

### Air sampling and analysis of heavy metals

The air sampling and analysis of heavy metals were based on the NIOSH method 7300^15^. After the weighing TSP, the remaining was also used to measure heavy metals, as described for TSP measurement. After sampling, PVC filter was folded four times into a microwave vessel, and then 4 ml of 70% nitric acid solution (Sigma Aldrich, MO, USA) was added. Samples were extracted using a PVC filter and PC(Polycarbonate) filter method stored in a library in a microwave (Model: MARS 6, CEM Corp., Matthews, NC, USA) for 2 times. The temperature was gradually raised to 200 °C for 20 min and then maintained for 20 min. The pressure was set to 800 psi, and the power to 900–1050 W. After this process, the vessel was cooled in the microwave for 20 min and at room temperature for 50 min. The extracted sample was diluted to 40 ml with 1% nitric acid solution, and quantitative analysis was performed using an inductively coupled plasma mass spectrometer (ICP-MS, Model: NexION 350D, Perkin Elmer Inc., Houston, TX, USA). The conditions show under the Table S3.

### Quality controls (QC)

Quality assurance and Quality controls (QA/QC) for heavy metals was performed. LODs of heavy metals was determined by triple standard deviations from seven replicates at the lowest level of the standard solution (1 to 20 μg/L), and a determination coefficient (r^2^) was more than 0.99 and it showed the linearity^16^. The values ​​are Cr 0.003, Mn 0.019, Co 0.003, Zn 0.017, Ni 0.013, Ag 0.014, Pb 0.006. Cd 0.008, Fe 0.005, Al 0.004, Ba 0.002, As 0.034, Sr 0.013, Na 0.004, Mg 0.032, Cu 0.010, K 0.037 µg/sample.

### Hood ventilation measurement

A thermal anemometer (TSI 9515, VelociCheck, TSI, USA) was used to measure the hood characteristics, ventilation volume (m^3^/s), and wind speed (m/s) according to distance. There are two types of thermal anemometers (one-way directional and omni-directional); this study used a one-way type. Figure S2 shows the overall shape of the hood; the workplace contained small, medium, and large hoods. Sampling and ventilation volume were measured at the medium and large hoods. The number 1 and 2 denotes medium and large hood, respectively. Five points on the hood were determined to measure ventilation and airflow. Point 1, 2 and 3 were controlled from the middle. Point 4 was controlled directly under the bath, and point 5 was measured for ventilation and airflow at a distance of 30 cm from the midpoint of points 1–4. The polishing process only had extra point.

### Data analysis

Air sample concentrations were expressed in terms of arithmetic mean (AM), standard deviation (SD), geometric mean (GM), geometric standard deviation (GSD), and median and range (min–max). The Shapiro–Wilk test confirmed that the TSP and heavy metals were log-normally distributed. Measured air concentrations of TSP and heavy metals in each process were compared using analysis of variance (ANOVA), and the Bonferroni method was used for post-hoc analysis. The date variations for TSP and heavy metals in each process were compared using ANOVA. Multivariate multiple linear regression was performed to find factors that affect TSP and heavy metals concentration. Response variables were TSP and heavy metals, and explanatory variables were workload, number of workers, and amount of ventilation volume. P < 0.05 was considered statistically significant. Statistical analysis was performed using R software v.3.6.3. (R Development Core Team, Vienna, Austria). Graphs were plotted using Sigmaplot 14.0 (Systat software, Inc, USA).

## Results

### TSP

**Figure 2:**
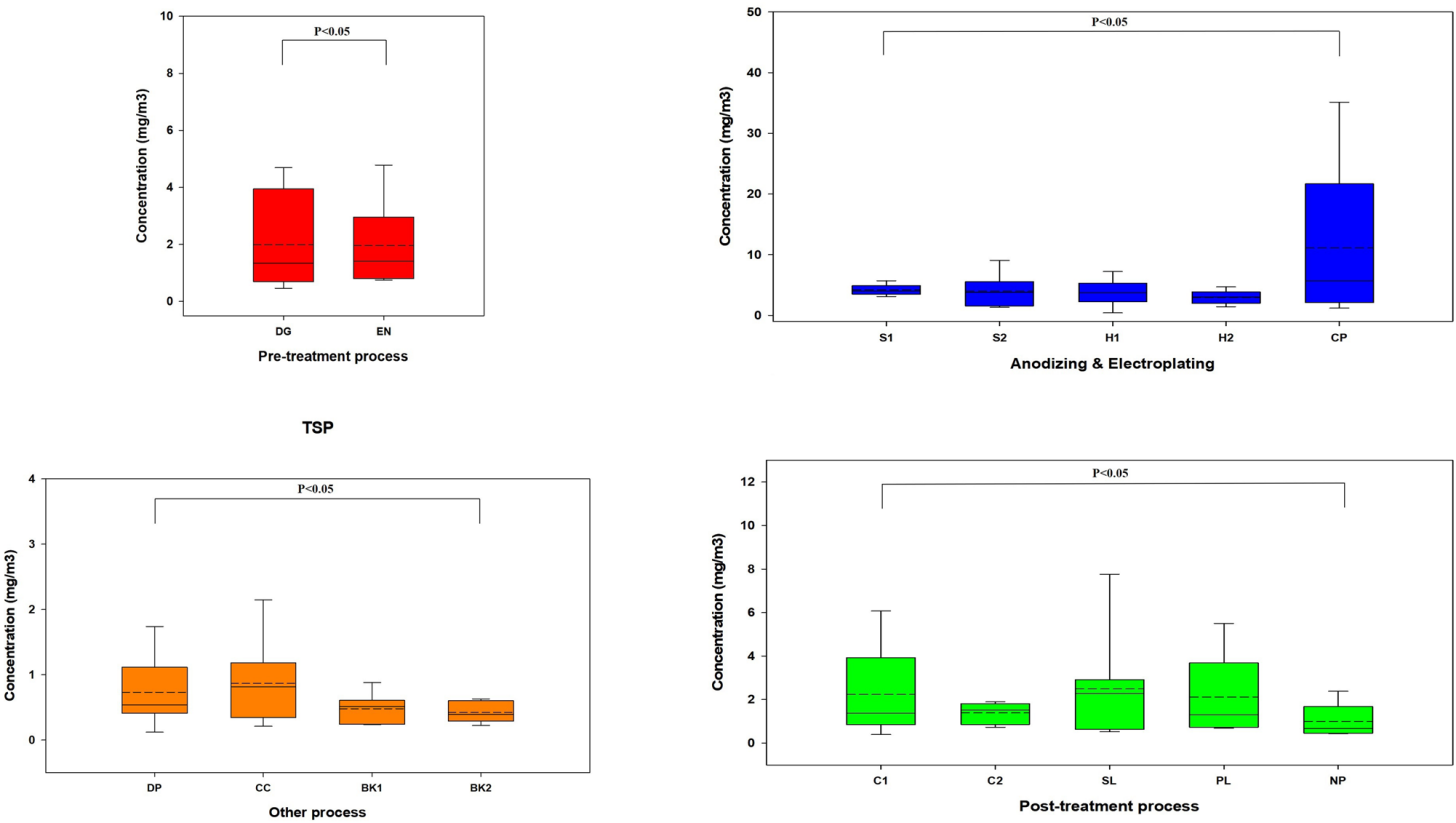
Bar plots of the TSP concentration air in all the processes. The TSP concentrations are arithmetic means. Values are median (line within box), mean (dotted line within box), 5th and 95th percentiles (bottom and top of box, respectively), minimum (lower bars on whisker), maximum (upper bars on whisker). *DG* Degreasing, *EN* Etching & Neutralization, *S1* Soft Anodizing_1, *S2* Soft Anodizing_2, *H1* Hard Anodizing_1, *H2* Hard Anodizing_2, *CP* Chromium plating, *C1* Coloring_1, *C2* Coloring_2, *SL* Sealing, *PL* Polishing, *NP* Non polishing, *DP* Drying & Packaging, *CC* Chromium cleaning, *B1* Blank (Plating & Anodizing), *B2* Blank (Drying & Packaging).

Table S4 and Table S5 show the TSP concentrations of all the process samples measured in the workplace. The highest GM concentrations of the samples were for Cr plating (6.15(GSD, 3.35) mg/m^3^). As a result of ANOVA test, GM concentrations of every process were statistically significant, showing that the process samples have different exposure aspects during product creation. Differences in GM concentrations and daily variation were statistically significant among the anodizing and electroplating, post-treatment, pre-treatment, and other processes. For the pre-treatment process, the average TSP concentration of degreasing was similar to that of etching and neutralization; however, the range was much higher than that of etching and neutralization. Anodizing and electroplating have similar soft and hard anodizing aspects, whereas Cr electroplating has a much higher concentration than all the processes. During the post-treatment process, the average TSP concentration of sealing has the highest average, whereas coloring and polishing have a wide concentration range. In the “other” processes, the TSP average concentration was generally lower. However, the drying and packaging and Cr cleaning processes have sufficient concentrations (Fig. 2).

### Heavy metals

**Figure 3:**
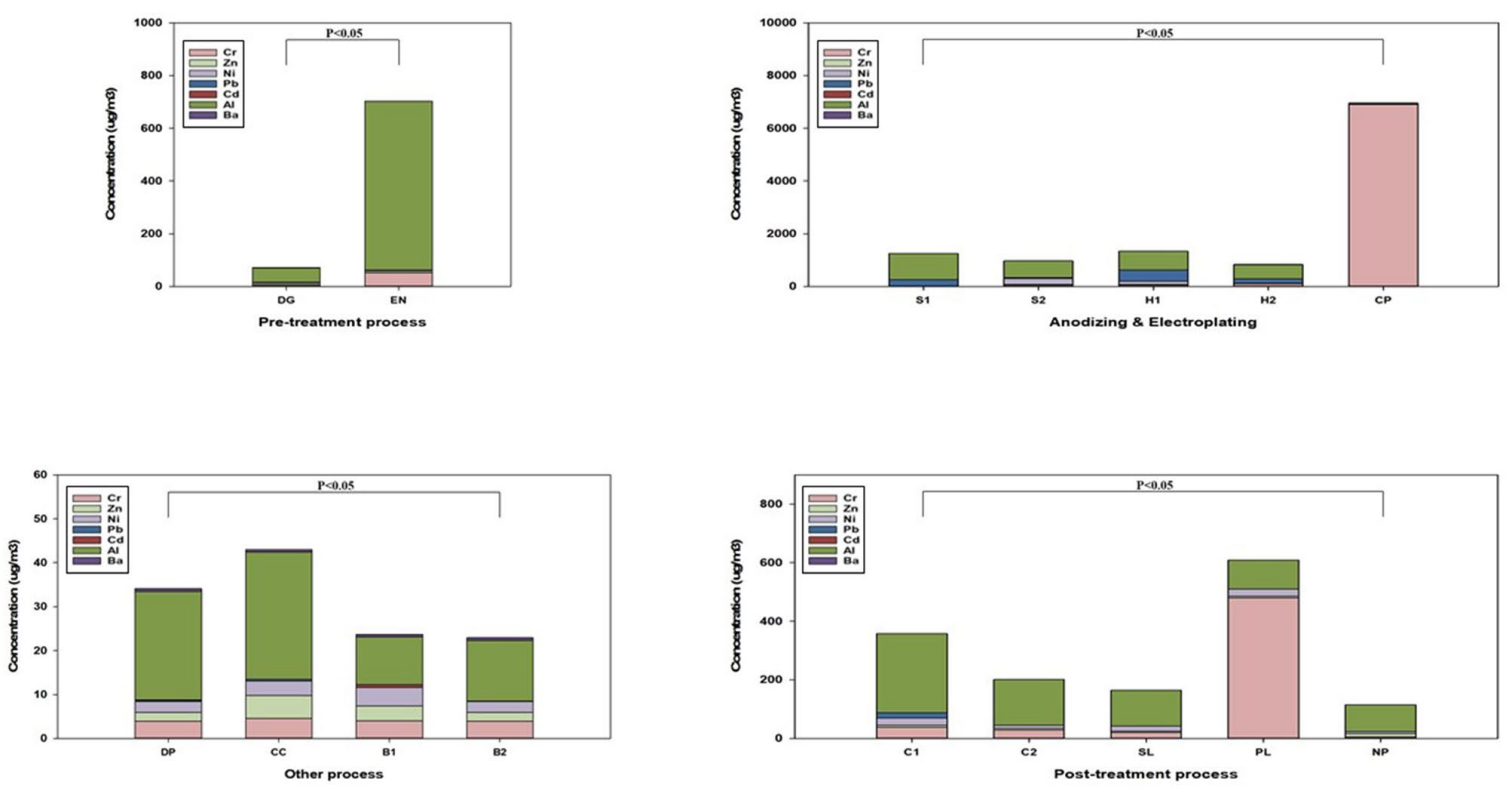
Heavy metals ratio in all the processes. The heavy metals concentrations are arithmetic means. There are a total of seven heavy metals. *Cr* Chromium, *Zn* Zinc, *Ni* Nickel, *Pb* Lead, *Cd* Cadmium, *Al* Aluminum, *Ba* Barium, *DG* Degreasing, *EN* Etching & Neutralization, *S1* Soft Anodizing_1, *S2* Soft Anodizing_2, *H1* Hard Anodizing_1, *H2* Hard Anodizing_2, *CP* Chromium plating, *C1* Coloring_1, *C2* Coloring_2, *SL* Sealing, *PL* Polishing, *NP* Non polishing, *DP* Drying & Packaging, *CC* Chromium cleaning, *B1* Blank (Plating & Anodizing), *B2* Blank (Drying & Packaging).

The heavy metal totals were analyzed with 17 types, including the existing seven (Cr, Mn, Zn, Ni, Pb, Cd, Al, and Ba) types and ten other types. Table S6, Table S7, Table S8, Table S9, Table S10, Table S11, Table S12 and Table S13 show the exposure to seven types of heavy metals in all workplace processes. GM of Cr during Cr plating was 1,859.66(6.65) µg/m^3^. GM of Cr during medium-sized soft anodizing, large-sized soft anodizing, medium-sized hard anodizing and large-sized hard anodizing were 52.94(17.71), 4.85(7.96), 151.40(11.09) and 85.52(4.82) µg/m^3^, respectively. Pb during Cr plating was 1.13(3.61) µg/m^3^. Al concentrations were high in most processes. GM of Al during medium-sized soft anodizing, large-sized soft anodizing, medium-sized hard anodizing, and large-sized hard anodizing were 528.97(6.80), 553.31(1.81), 545.98(2.75), and 520.79(1.34) µg/m^3^, respectively. Differences in GM concentrations and daily variation of the GMs of heavy metals were statistically significant. When the proportion of heavy metals was confirmed, the analysis was performed with seven basic heavy metals, excluding the 10 other heavy metals. Pre-treatment processes confirmed that Al occupied the largest proportion of heavy metals, followed by Cr, and occupied most of the anodizing and electroplating processes; for Cr plating, however, Cr occupied the largest proportion. The post-treatment process revealed that the Cr concentration occupied a high rate while polishing. In other processes, Al occupied the highest rate, and Ni occupied the highest rate in the drying and packaging processes (Fig. 3).

### Relationship between TSP and heavy metals

**Figure 4:**
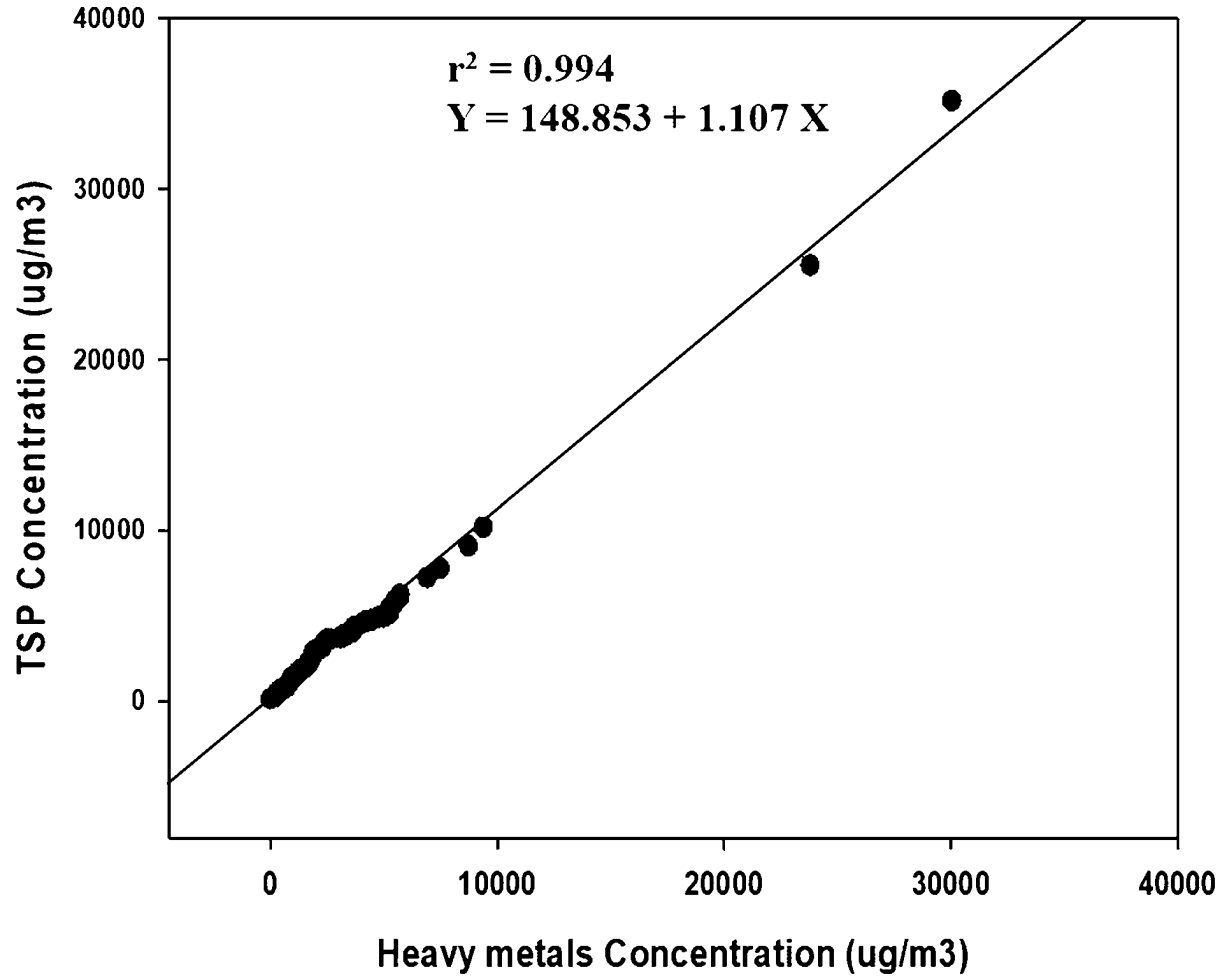
Distribution of heavy metals and TSP. All the concentrations are arithmetic means (N = 107).

**Figure 5:**
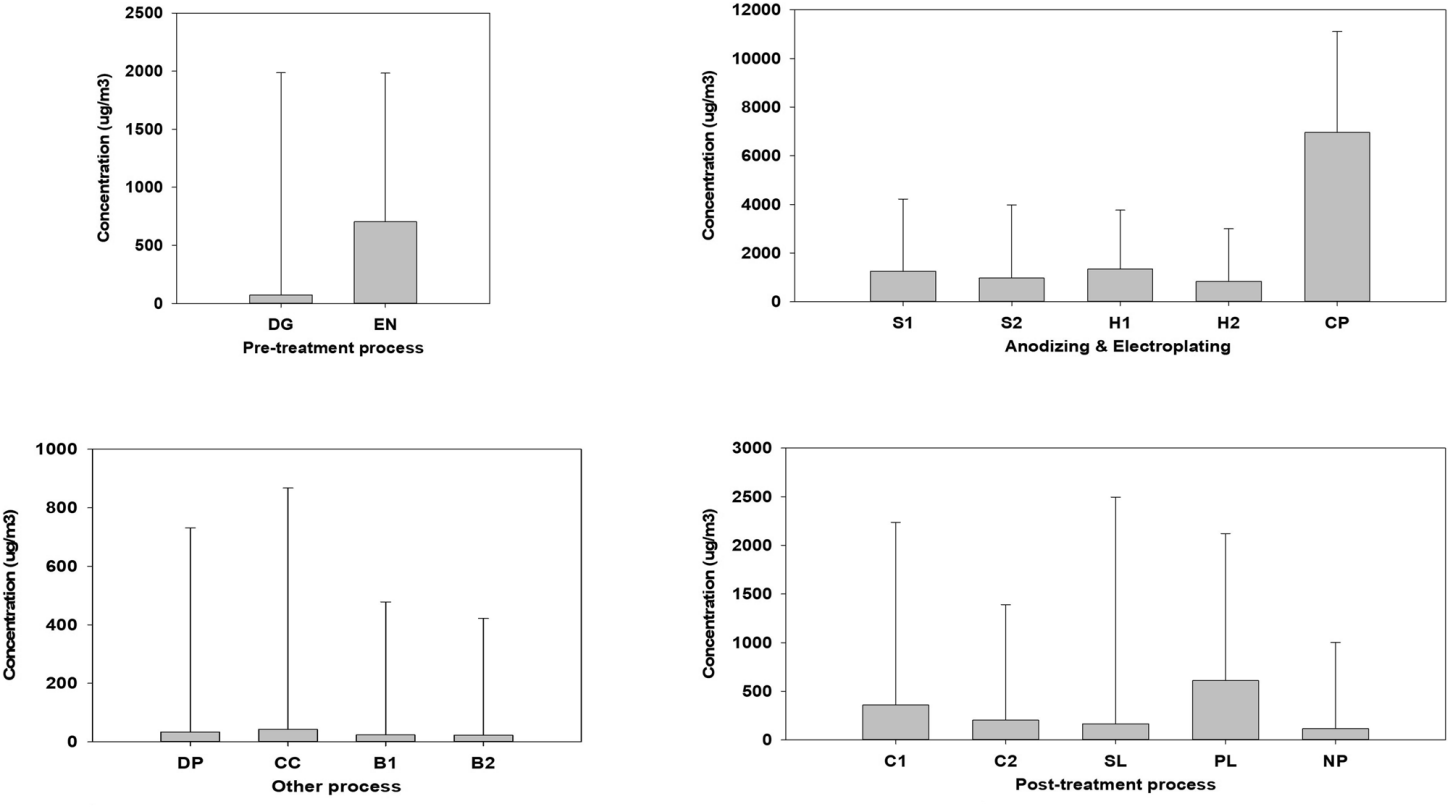
Ratio of heavy metals and other substances in all the processes. The heavy metals concentrations are arithmetic means for a total of seven heavy metals. Error bar denote other substances. *DG* Degreasing, *EN* Etching & Neutralization, *S1* Soft Anodizing_1, *S2* Soft Anodizing_2, *H1* Hard Anodizing_1, *H2* Hard Anodizing_2, *CP* Chromium plating, *C1* Coloring_1, *C2* Coloring_2, *SL* Sealing, *PL* Polishing, *NP* Non polishing, *DP* Drying & Packaging, *CC* Chromium cleaning, *B1* Blank (Plating & Anodizing), *B2* Blank (Drying & Packaging).

Figure 4 shows the correlation distribution between TSP and heavy metals (N = 107) concentrations. The coefficient of determination r^2^ was 0.994, indicating a strong relationship. The ratio of seven kinds of heavy metals causing adverse health effects relative to the total measured substances in the air was not relatively high. The Cr concentration accounted for 62.65% in degreasing, drying and packaging, and Cr cleaning and for 20–40% in soft and hard anodizing (Fig. 5).

### Ventilation

The workplace was equipped with a hood for every process, and the total suction flow rate for all the processes was 600 m^3^/s. The hood had a total of four slots, and the polishing process involved a total of five slots. Table S14 and Table S15 show the total ventilation volume for each slot in all the processes; the average ventilation volume for all hoods ranged from 1.20–4.98 m^3^/s. Slot 3 is designed to have the highest ventilation volume of the hood, and slot 4 is designed to be the next highest, with suction directly below. For the pre-treatment process, the average degreasing (1.74 m^3^/s) and etching & neutralization (1.77 m^3^/s) were ventilated. For anodizing and electroplating, medium-sized soft anodizing was 1.76 m^3^/s, and large soft anodizing was 2.14 m^3^/s. The medium-sized and large-sized hard anodizing had average ventilation volumes of 2.12 and 2.33 m^3^/s, respectively. Ventilation volume for Cr plating was 2.94 m^3^/s and was the largest than in any other processes. For the post-treatment process, the medium-sized and large-sized colorings are 1.22 and 1.31 m^3^/s, respectively. The ventilation of the hood 30 cm away from the bath was very low. The 30 cm away inhalation volume was 0.1 times lower than the average ventilation volume.

### Regression analysis for TSP and heavy metals

Multiple linear regression analysis was conducted by setting TSP and heavy metals (N = 107) as response variables, and environmental factors were expected to affect the concentrations of TSP and heavy metals in the workplace as explanatory variables (Table 2). Regression model of TSP analysis with three variates was statistically significant. Ventilation volume at a distance of 30 cm from the hood, machine operating time, and workload affected TSP concentrations. The most influential factor on TSP in the workplace was ventilation volume at a distance of 30 cm from the hood. TSP coefficient decreased by 0.94 as ventilation volume standard deviation (SD) 1 increased. TSP coefficient increased by 0.62 as machine operating time SD 1 increased. As workload SD 1 increased, TSP coefficient increased by 0.18. From statistical analysis of heavy metals, the regression model with three variates was statistically significant. Ventilation volume at a distance of 30 cm from the hood, machine operating time, and workload affected heavy metals concentrations. The most influential factor on heavy metals in the workplace was ventilation volume at a distance of 30 cm from the hood; heavy metals coefficient decreased by 0.93 as ventilation volume SD 1 increased. Heavy metals coefficient increased by 0.63 as machine operating time SD 1 increased, and workload SD 1 increased as the heavy metals coefficient increased by 0.13.

**Table 2:** Multiple linear regression on TSP and heavy metals concentrations and environmental factors.

Variables	TSP				Heavy metals			
	R (Correlation)	$\beta$** ($\beta$***)	t –value *	F	R (Correlation)	$\beta$**($\beta$***)	t –value *	F
Hood 30 cm distance ventilation	0.49	− 0.94 (− 1,031.37)	− 4.55	22.31	0.38	− 0.93 (− 930.34)	− 4.51	20.78
Machine operation time	0.28	0.62 (14.97)	2.92	14.02	0.27	0.63 (13.50)	2.96	12.92
Workload	0.11	0.18 (13.44)	2.05	10.99	0.13	0.13 (12.12)	2.38	10.82

*p < 0.05.

**Standardization factor.

***Non-standardization factor.

## Discussion

This workplace conducts work environment measurements conducted by external work environment measurement agencies twice a year. The agencies can evaluate intensive personal exposure, but cannot determine the primary cause of the occurrence of harmful substances. Most acids (hydrochloric acid, nitric acid, phosphoric acid, and hydrofluoric acid) were not detected. Cr (VI) was also not detected, but Ni, sulfuric acid, and sodium hydroxide were 0.001–0.003, 0.021–0.035 and 0.349–0.453 µg/m^3^. This study conducted a two-week measurement to reduce date variation to improve the limit of one-time work environment measurements.

### Aspects of TSP and heavy metals in workplace

The exposure values of the anodizing and electroplating processes, which handle more heavy metals were higher. The concentrations of some types of heavy metals in all the processes were below the OEL of Korea; however, the Cr processes exceeded the OEL of Korea, which far exceeded the OEL of Korea (Cr(VI) = 10 µg/m^3^). Some Zn processes exceeded the OEL of Korea, whereas others had values close to those allowed in the working environment. The levels of Pb in anodizing and electroplating processes were higher than the OEL of Korea (Pb = 50 µg/m^3^). Cd, Al, and Ba did not exceed the OEL of Korea (Cd = 10 µg/m^3^, Al(fume) = 5,000 µg/m^3^. Ba = 500 µg/m^3^). However, in the case of Al, high concentrations were medium-sized and large-sized soft anodizing, and medium-sized and large-sized hard anodizing. Concentrations were also relatively high in coloring and sealing. The principal factor affecting the concentration was ventilation volume, following machine operation time and workload. The ventilation volume of the hood in the workplace was low; thus, the suction flow rate of the hood had to be improved.

According to Previous research, measured concentrations of TSP, Cr, Ni, Cu, Mn, Co, and Pb in the metal finishing industry were 0.32 ± 0.11, 109.9E^−6^ ± 38.4E^−6^, 81.3E^−6^ ± 89.5E^−6^, 274.2E^−6^ ± 118.4E^−6^, 206.2E^−6^ ± 173.4E^−6^, 73.3E^−6^ ± 44.8E^−6^, and 69.9E^−6^ ± 44.5E^−6^ mg/m^3^. Heavy metals concentrations were very low, whereas TSP levels were relatively high^17^. When checking the distribution of sample concentrations in Cr and Cr (VI) by samples for 10 plants (n = 48), arithmetic mean (AM) range of total and Cr (VI) concentrations were 0.89–523.7 and 0.09–113.2 µg/m^3^, respectively. Moreover, degreasing had the highest GM 21.6 µg/m^3^, followed by the plating process (13.4 µg/m^3^) and “other” process (2.36 µg/m^3^). Cr (VI) concentration with plating had the highest GM (4.15 µg/m^3^), followed by degreasing (1.86 µg/m^3^) and “other” process (1.28 µg/m^3^). Total Cr and Cr (VI) showed a high correlation of 0.91^18^. In the soil around the electroplating, Cr, Cu, Pb, Zn, and Cd were detected at concentrations of 22.7–453, 9.51–120, 18–108, 24.1–143, and 0.130–0.850 mg/kg, respectively. Therefore, heavy metals in air can be detected at high concentrations in the surrounding environment for the electroplating plant^19^. Individual samples were measured for Ni and Cr at three factories in the electroplating industry, and the concentrations in air and urine were checked. Arithmetic mean (AM) and Standard deviation(SD) of Ni and Cr were 7.4 ± 6.4 and 3.0 ± 3.6 µg/m^3^ in the air. Additionally, creatinine concentrations were 30.1 ± 19.5 and 76.3 ± 54.5 µg/g in urine. Individual exposure was not very high in air, and the correlation with urine concentrations was weak^20^. The factors influencing individual Cr concentration in Cr plating factories were confirmed by regression analysis. The exposure coefficients for wearing/not wearing gloves, smoking, and exposure after recently had a skin disease were 1.85, 0.33, and 0.45, respectively. Washing hands before going to the bathroom reduced the Cr concentration coefficient to − 0.32^21^.

### Particle size of heavy metals in surface treatment

At a metal finishing industry, PM2.5 of Cr, Ni, Cu, Mn, Co, and Pb were 86.7E^-6^ ± 17.3E^-6^, 65.3E^-6^ ± 88.9E^-6^, 144.5E^-6^ ± 143.6E^-6^, 46.2E^-6^ ± 25.8E^-6^, 35.9E^-6^ ± 22.0E^-6^, and 205.1E^-6^ ± 95.1E^-6^, respectively. Additionally, PM1 of Cr, Ni, Cu, Mn, Co, and Pb were 73.2 E^-6^ ± 11.7 E^-6^, 55.7 E^-6^ ± 78.0 E^-6^, 102.1 E^-6^ ± 103.5 E^-6^, 39.6 E^-6^ ± 20.9 E^-6^, 23.8 E^-6^ ± 22.6 E^-6^, and 161.6 E^-6^ ± 65.6 E^-6^ mg/m^3^, respectively. Although smaller particle size resulted in lower concentration, the adverse effect on health was greater at smaller particle sizes. Additionally, finer particles can move the alveoli of the lungs^17,22^. The diameter distribution of Cr (VI) less than 10 µm in the Cr plating plant was investigated, and the mass median diameters of Cr particles in the two electroplating tanks were 5.11 µm and 6.38 µm, respectively. The Cr (VI) diameter distribution in the general manufacturing industry was 1.67 µm. Therefore, the electroplating tank had a relatively high mass distribution^23^.

### Limitations

The ventilation flow rate of the designed overall hood is 600 m^3^/s. On the other hand, the ventilation flow rate of individual hoods is relatively low. In other words, it can be inferred that the hood or duct is poorly engineered or maintained due to aging. The reason why the deviation increased in high concentration level is because we mainly evaluated TSP. Large particles are measured due to leakage from the ventilation problem, and high outliers were observed.

Calculating the workload of the detailed process the precise amounts of chemical substances used in the electrolytes is difficult, because most materials are trade secrets. TSP was investigated in all the processes while making metal products. The quantitative composition of heavy metals particle size PM0.3 to PM10 using a real-time measuring instrument (Aerotrak Particle counter, TSI, USA) found that smaller particle sizes correlate to higher quantitative composition concentration in all the processes; among these, a higher quantitative composition concentration was measured as the particle size decreased in degreasing, Cr plating, and Ni plating^24^.

Checking the concentration of dust by particle size to provide information that can explain the extent of inhalation of smaller particles into the human body is necessary. A study for sampling nanoparticle size in electroplating revealed that the generation of nanoparticles were 64,327 particles/cm^3^ with a passivating bath process and 33,249 particles/cm^3^ without a passivating bath process based on 8 h TWA (Time-Weighted Average). Additionally, 5,645 and 4,947 particles/cm^3^ were generated when working with and without the rotary abraser bath process, respectively. Thus, further research on nanoparticles is important^25^.

## Conclusions

Most of the work environment measurement agency data at this workplace were undetected. Firstly, identifying the fundamental cause of the high concentration in the processes is difficult. Work environment measurement system is intensively focused on personal exposure. Obtaining a sufficient number of samples N of data is hard. Therefore, to confirm the reliability of work environment measurement system, conducting simultaneous personal and area exposure measurements would improve the results. suction flow rate was exceptionally low and difficult to collect when measured from a distance of 30 cm.

The ventilation volume of the hood should be increased to make the total volume similar to 600 m^3^/s. In addition, ventilation plans must be taken to reduce indoor pollutants by adding an external ventilation system. Therefore, improving the suction flow rate of the hood is necessary.Few studies have measured and evaluated exposure to TSP and heavy metals related to anodizing and electroplating. In this study, we have confirmed that the exceptionally high concentrations of TSP and heavy metals are primarily caused by low ventilation suction flow rates. Concentrations were similar among pre-treatment and anodizing processes, especially high in the Cr plating process, and primarily high in the coloring and polishing processes among the post-treatment process. Concentrations in other processes remained relatively high. Although in most processes, the Al concentration was the highest, Cr concentration was high in the Cr plating and polishing processes. This study provides data on the risk of exposure for anodizing and electroplating and improves the reliability of work environment measurement data, thereby improving health through the need for fundamental exposure. Since the amount of work was also different every day for each process, this showed a large variation for each sample. The reason why the ventilation amount differs for each process is primarily due to the size of the hood. The amount of ventilation may vary depending on the size of the hood. Secondly, because of the aging of the hood, foreign substances in the duct connected to the hood and foreign substances in the hood slot have been formed over a long period of use, but they have not been cleaned or maintained. It can be a data that can raise the problem of a one-time work environment measurement agency data. Room humidity, temperature, and product characteristics were predicted the variables affecting date concentration.

## Supplementary Information

Supplementary Information.

## Data Availability

Additional information associated with this article can be found in the supplementary data.
