# Supplementary material for: Evaluation of airborne total suspended particulates and heavy metals in anodizing and electroplating surface treatment process
Source: Sci Rep. 2021 Nov 18;11:22537. doi: 10.1038/s41598-021-01577-9 (PMC8602259; doi:10.1038/s41598-021-01577-9)
Supplement: Supplementary file 1 — Supplementary Information. [file 41598_2021_1577_MOESM1_ESM.docx]

# **Supplementary Materials**

**Table of Contents**

**Table S1.** pH condition, operation voltage and working environment in the processes

**Table S2.** Workload and worker factors

**Table S3.** ICP/MS conditions for analyzing heavy metals

**Table S4.** TSP in pre-treatment & surface treatment process

**Table S5.** TSP in post-treatment process

**Table S6.** Heavy metals in pre-treatment process

**Table S7.** Heavy metals in surface treatment-soft anodizing process

**Table S8.** Heavy metals in surface treatment-hard anodizing process

**Table S9.** Heavy metals in surface treatment-chromium plating process

**Table S10.** Heavy metals in post treatment-coloring process

**Table S11.** Heavy metals in post treatment-sealing process

**Table S12.** Heavy metals in post treatment-polishing process

**Table S13.** Heavy metals in post treatment-packaging & chromium cleaning process

**Table S14.** Hood ventilation flow rate in pre-treatment & surface treatment process

**Table S15.** Hood ventilation flow rate in post-treatment process

**Fig. S1.** Anodizing and electroplating schematic and sample location 2: the icons are the sampling locations (orange star: other processes).

**Note**: Workplace(31m×14m)

**Fig. S2.** Schematic diagram of hood and bath.

**Table S1.** pH condition, operation voltage and working environment in the processes

| **pH** | | | | | | | |
| --- | --- | --- | --- | --- | --- | --- | --- |
|  | | **Coloring 1^*^** | | **Coloring 2^**^** | | **Sealing** | |
| **pH** | | 5 - 6 | | 5 - 6 | | 5 - 6 | |
|  |  | |  |  |  | |  |
| **Voltage** | | | | | | | |
|  | **Soft anodizing 1^*^** | | **Soft anodizing 2^**^** | **Hard anodizing 1^*^** | **Hard anodizing 2^**^** | | **Chromium plating** |
| **operation voltage(V)** | 10 - 17 | | 10 - 17 | 10 - 36 | 10 - 36 | | 10 - 36 |
|  |  | |  |  |  | |  |
| **working environment** ^a^ | | | | | | | |
|  | | | **Range** | | **Mean** | | |
| **temperature ℃** | | | 22.90 – 34.10 | | 28.59 | | |
| **humidity %** | | | 36.6 – 65.4 | | 56.58 | | |

^*^ 1= Medium size of bath

^**^ 2=Large size of bath

^a^ : The date variation of average temperature and humidity at workplace

**Table S2.** Workload and worker factors

| **Days** | **Workload**  **(piece)** | **Worker** |
| --- | --- | --- |
| **1 day** | 3,000 | 16 |
| **2 day** | 4,500 | 18 |
| **3 day** | 2,500 | 18 |
| **4 day** | 3,400 | 17 |
| **5 day** | 4,300 | 13 |
| **6 day** | 5,800 | 15 |
| **7 day** | 3,200 | 16 |
| **8 day** | 3,500 | 15 |

**Table S3.** ICP/MS conditions for analyzing heavy metals

| **Parameter** | **Analytical conditions** |
| --- | --- |
| **Nebulizer** | Concentric glass nebulizer |
| **RF generator(W)** | Power output: 500 W – 1,600 W |
| **Spray chamber** | Glass cyclonic spray chamber |
| **Hyper-Skimmer cone (mm)** | Aluminum alloy 1.0 |
| **Argon flow rate** | |
| **Plasma gas (L/min)** | 18.00 |
| **Auxiliary gas (L/min)** | 1.20 |
| **Nebulizer gas (L/min)** | 0.96 |
| **Sampler cone (mm)** | Nickel 1.0 |
| **Skimmer cone (mm)** | Nickel 0.9 |
| **Vacuum** | |
| **Inter face(torr)** | < 2 x 10^-6^ |
| **Quadrapole(torr)** | < 3 x 10^-8^ |
| **Data acquisition** | Peak hopping, 1 reading 20 sweep, 3 replicates |
| **Measurement mode** | Quantification mode |

**Table S4.** TSP in pre-treatment & surface treatment process

| **Process (N=53)** | **Concentration (mg/㎥)** | | | |
| --- | --- | --- | --- | --- |
|  | **Mean**±**SD** | **GM(GSD)** | **Range (Median)** | **P-Value** |
| **Degreasing (N=8)** | 1.99±1.71 | 1.44(2.36) | 0.46-4.70 (1.34) | *p <0.05^a^* |
| **Etching & Neutralization (N=5)** | 1.95±1,.45 | 1.56(2.05) | 0.76 – 4.78 (1.42) |  |
| **Soft Anodizing_1* (N=8)** | 4.22±8.96 | 4.14(1.23) | 3.13 – 5.72 (4.02) | p <0.05a |
| **Soft Anodizing_2** (N=8)** | 3.98±2.63 | 3.25(2.01) | 1.32 – 9.07 (3.81) |  |
| **Hard Anodizing_1* (N=8)** | 3.76±2.10 | 2.98(2.42) | 0.42 – 7.22 (3.72) |  |
| **Hard Anodizing_2** (N=8)** | 3.00±1.14 | 2.80(1.51) | 1.43 – 4.69 (3.01) |  |
| **Chromium plating (N=8)** | 11.10±12.47 | 6.15(3.35) | 1.20 – 35.15 (5.68) |  |

* 1= Medium size of bath

** 2=Large size of bath

dotted line: group of process

**Abbreviation**: SD=Standard Deviation, GM=Geometric Mean, GSD=Geometric Standard Deviation

^a^: The GM of TSP concentration difference among each work process(ANOVA test)

**Table S5.** TSP in post-treatment process

| **Process (N=54)** | **Concentration (mg/㎥)** | | | |
| --- | --- | --- | --- | --- |
|  | **Mean**±**SD** | **GM(GSD)** | **Range (Median)** | **P-Value** |
| **Coloring_1**^*^ **(N=8)** | 2.24±2.00 | 1.58(2.48) | 0.40 – 6.07 (1.38) | *p <0.05^a^* |
| **Coloring_2**^**^ **(N=8)** | 1.39±0.46 | 1,31(1.46) | 0.73 – 1.90 (1.53) |  |
| **Sealing (N=8)** | 2.49±2.37 | 1,71(2.60) | 0.53 - 7.76 (2.29) |  |
| **Polishing (N=7)** | 2.12±1.81 | 1.60(2.21) | 0.68 – 5.51 (1.31) |  |
| **Non polishing (N=7)** | 1.00±0.74 | 0.82(1.92) | 0.44 – 2.38 (0.67) |  |
| **Drying & Packaging (N=8)** | 0.73±0.5294 | 0.57(2.27) | 0.12 – 1.74 (0.54) |  |
| **Chromium cleaning (N=8)** | 0.87±0.63 | 0.68(2.17) | 0.21 – 2.15 (0.81) |  |

* 1= Medium size of bath

** 2=Large size of bath

dotted line: group of process

**Abbreviation**: SD=Standard Deviation, GM=Geometric Mean, GSD=Geometric Standard Deviation

^a^: The GM of TSP concentration difference among each work process(ANOVA test)

**Table S6.** Heavy metals in pre-treatment process

| **Process**  **(N=13)** |  | **heavy metals (μg/㎥)** | | | | | | |
| --- | --- | --- | --- | --- | --- | --- | --- | --- |
|  |  | **Cr** | **Zn** | **Ni** | **Pb** | **Cd** | **Al** | **Ba** |
| **Degreasing** ^a^  **(N=8)** | Mean±SD | 6.14±3.52 | 6.38±4.16 | 3.95±3.57 | 0.52±0.27 | 0.05±0.06 | 58.91± 40.31 | 1.14±1.57 |
|  | GM(GSD) | 5.49(1.61) | 5.45(1.77) | 3.03(2.05) | 0.45(1.90) | 0.03(2.86) | 45.60(2.36) | 0.73(2.36) |
|  | Range (Median) | 2.89 – 14.13 (5.26) | 3.19 - 13.59 (4.46) | 1.70 - 11.83 (2.10) | 0.14 - 0.93 (0.57) | 0.01 - 0.17 (0.02) | 7.81 - 128.95 (48.27) | 0.29 - 4.99 (0.67) |
| **Etching & Neutralization** ^a^  **(N=5)** | Mean±SD | 51.10±38.00 | 6.69±8.34 | 3.35±3.88 | 0.49±0.35 | 0.03±0.03 | 72.86±25.77 | 0.76±0.67 |
|  | GM(GSD) | 33.16(3.67) | 4.23(2.65) | 2.33(2.32) | 0.38(2.43) | 0.03(2.04) | 69.69(1.39) | 0.62(1.93) |
|  | Range (Median) | 3.71 - 105.18 (46.81) | 1.99 - 21.50 (3.96) | 1.28 - 10.28 (1.71) | 0.11 - 0.98 (0.54) | 0.01 - 0.08 (0.03) | 47.33 - 115.93 (65.05) | 0.39 - 1.96 (0.47) |

^a^ Significant difference (p<0.05) of pre-treatment process(ANOVA test)

**Abbreviation**: SD=Standard Deviation, GM=Geometric Mean, GSD=Geometric Standard Deviation, Cr=Chromium(Ⅵ), Zn=Zinc, Ni=Nickel, Pb=Lead, Cd=Cadmium, Al=Aluminum, Ba=Barium

**Table S7.** Heavy metals in surface treatment-soft anodizing process

| **Process**  **(N=16)** |  | **heavy metals (μg/㎥)** | | | | | | |
| --- | --- | --- | --- | --- | --- | --- | --- | --- |
|  |  | **Cr** | **Zn** | **Ni** | **Pb** | **Cd** | **Al** | **Ba** |
| **Soft**  **Anodizing_1^*^** ^b^  **(N=8)** | Mean±SD | 7.35±2.54 | 5.92±3.05 | 4.42±4.83 | 244.23±273.60 | 0.02±0.01 | 989.21±569.23 | 0.77±0.30 |
|  | GM(GSD) | 6.99(1.39) | 5.14(1.81) | 3.10(2.28) | 52.94(17.71) | 0.02(1.61) | 528.97(6.80) | 0.70(1.60) |
|  | Range (Median) | 4.31 - 11.44 (6.54) | 2.29 - 9.31 (5.87) | 1.33 - 15.78 (2.76) | 0.31 - 694.01 (144.80) | 0.01 - 0.03 (0.02) | 5.57 – 1,680.04 (1,107.71) | 0.30 - 1.18 (0.85) |
| **Soft Anodizing_2**** ^b^  **(N=8)** | Mean±SD | 63.22±102.54 | 7.51±3.76 | 228.77±625.47 | 32.59±56.90 | 0.03±0.02 | 634.30±322.46 | 0.90±0.74 |
|  | GM(GSD) | 20.70(4.79) | 6.45(1.92) | 7.91(11.57) | 4.85(7.96) | 0.02(2.07) | 553.31(1.81) | 0.67(2.27) |
|  | Range (Median) | 3.44 - 294.71 (9.88) | 1.73 - 12.44 0(7.71) | 1.10 - 176.47 (3.95) | 0.79 - 133.62 (2.02) | 0.01 - 0.06 (0.03) | 192.40 – 1,097.18 (614.65) | 0.25 - 2.29 (0.56) |

^*^ 1= Medium size of bath

^**^ 2=Large size of bath

^b^ Significant difference (p<0.05) of surface treatment process(ANOVA test)

**Abbreviation**: SD=Standard Deviation, GM=Geometric Mean, GSD=Geometric Standard Deviation, Cr=Chromium(Ⅵ), Zn=Zinc, Ni=Nickel, Pb=Lead, Cd=Cadmium, Al=Aluminum, Ba=Barium

**Table S8.** Heavy metals in surface treatment-hard anodizing process

| **Process**  **(N=16)** |  | **heavy metals (μg/㎥)** | | | | | | |
| --- | --- | --- | --- | --- | --- | --- | --- | --- |
|  |  | **Cr** | **Zn** | **Ni** | **Pb** | **Cd** | **Al** | **Ba** |
| **Hard Anodizing_1^*^** ^b^  **(N=8)** | Mean±SD | 59.09±81.91 | 8.68±7.18 | 125.63±331.32 | 422.00±384.57 | 0.02±0.01 | 720.22±406.06 | 0.74±0.45 |
|  | GM(GSD) | 23.76(4.51) | 6.88(2.01) | 7.01(10.88) | 151.40(11.09) | 0.02(1.41) | 545.98(2.75) | 0.62(2.05) |
|  | Range (Median) | 4.17 - 243.31 (20.92) | 2.95 - 25.09 (6.96) | 0.88 - 945.16 (2.96) | 0.65 – 1,008.00 (314.62) | 0.01 - 0.04 (0.02) | 52.70 – 1,323.33 (694.99) | 0.13 - 1.69 (0.67) |
| **Hard Anodizing_2^**^** ^b^  **(N=8)** | Mean±SD | 99.10±212.84 | 6.94±3.67 | 12.68±16.59 | 169.68±147.95 | 0.03±0.03 | 541.11±168.11 | 0.87±0.71 |
|  | GM(GSD) | 18.19(6.27) | 6.16(1.68) | 5.69(4.10) | 85.52(4.82) | 0.02(2.07) | 520.79(1.34) | 0.71(1.88) |
|  | Range (Median) | 3.35 - 617.45 (8.07) | 3.27 - 13.68 (6.21) | 1.00 - 48.77 (6.16) | 6.53 - 437.51 (169.17) | 0.01 - 0.08 (0.02) | 351.73 - 881.67 (514.37) | 0.39 - 2.51 (0.61) |

^*^ 1= Medium size of bath

^**^ 2=Large size of bath

^b^ Significant difference (p<0.05) of surface treatment process(ANOVA test)

**Abbreviation**: SD=Standard Deviation, GM=Geometric Mean, GSD=Geometric Standard Deviation, Cr=Chromium(Ⅵ), Zn=Zinc, Ni=Nickel, Pb=Lead, Cd=Cadmium, Al=Aluminum, Ba=Barium

**Table S9.** Heavy metals in surface treatment-chromium plating process

| **Process**  **(N=8)** |  | **heavy metals (μg/㎥)** | | | | | | |
| --- | --- | --- | --- | --- | --- | --- | --- | --- |
|  |  | **Cr** | **Zn** | **Ni** | **Pb** | **Cd** | **Al** | **Ba** |
| **Chromium**  **plating** ^b^  **(N=8)** | Mean±SD | 6,903.84±10,402.75 | 8.07±5.38 | 6.89±4.95 | 2.30±2.87 | 0.04±0.03 | 33.63±19.45 | 0.96±0.58 |
|  | GM(GSD) | 1,859.66(6.65) | 6.83(1.82) | 5.54(2.03) | 1.13(3.61) | 0.03(1.98) | 27.05(2.22) | 0.74(2.35) |
|  | Range (Median) | 155.35 - 28193.12 (1,744.05) | 3.48 - 19.33 (5.94) | 2.29 - 16.55 (6.15) | 0.21 - 7.56 (0.94) | 0.01 - 0.08 (0.03) | 7.01 - 67.78 (35.78) | 0.19 - 1.63 (0.95) |

^b^ Significant difference (p<0.05) of surface treatment process(ANOVA test)

**Abbreviation**: SD=Standard Deviation, GM=Geometric Mean, GSD=Geometric Standard Deviation, Cr=Chromium(Ⅵ), Zn=Zinc, Ni=Nickel, Pb=Lead, Cd=Cadmium, Al=Aluminum, Ba=Barium

**Table S10.** Heavy metals in post treatment-coloring process

| **Process**  **(N=16)** |  | **heavy metals (μg/㎥)** | | | | | | |
| --- | --- | --- | --- | --- | --- | --- | --- | --- |
|  |  | **Cr** | **Zn** | **Ni** | **Pb** | **Cd** | **Al** | **Ba** |
| **Coloring_1^*^** ^c^  **(N=8)** | Mean±SD | 38.73±50.09 | 6.62±2.81 | 24.93±40.97 | 17.46±47.90 | 0.03±0.02 | 269.45±241.15 | 0.75±0.62 |
|  | GM(GSD) | 18.34(6.27) | 6.17(1.48) | 6.94(5.40) | 0.89(8.63) | 0.03(1.72) | 190.66(2.52) | 0.55(2.48) |
|  | Range (Median) | 3.36 - 144.39 (10.56) | 4.01 - 11.88 (5.13) | 1.25 - 112.73 (3.40) | 0.14 – 136.00 (0.59) | 0.01 - 0.07 (0.03) | 51.05 - 796.83 (219.98) | 0.10 - 2.07 (0.54) |
| **Coloring_2^**^** ^c^  **(N=8)** | Mean±SD | 28.77±40.00 | 5.39±2.53 | 10.51±17.04 | 0.71±0.75 | 0.07±0.11 | 155.51±141.12 | 0.62±0.53 |
|  | GM(GSD) | 13.16(3.72) | 4.89(1.62) | 4.27(3.88) | 0.50(2.26) | 0.04(2.63) | 126.15(1.83) | 0.48(2.16) |
|  | Range (Median) | 3.91 - 108.99 (6.78) | 2.08 – 10.00 (4.91) | 1.12 - 51.09 (2.51) | 0.23 - 2.42 (0.43) | 0.02 - 0.33 (0.02) | 72.84 - 499.91 (111.99) | 0.12 - 1.84 (0.46) |

^*^ 1= Medium size of bath

^**^ 2=Large size of bath

^c^ Significant difference (p<0.05) of post-treatment process(ANOVA test)

**Abbreviation**: SD=Standard Deviation, GM=Geometric Mean, GSD=Geometric Standard Deviation, Cr=Chromium(Ⅵ), Zn=Zinc, Ni=Nickel, Pb=Lead, Cd=Cadmium, Al=Aluminum, Ba=Barium

**Table S11.** Heavy metals in post treatment-sealing process

| **Process**  **(N=8)** |  | **heavy metals (μg/㎥)** | | | | | | |
| --- | --- | --- | --- | --- | --- | --- | --- | --- |
|  |  | **Cr** | **Zn** | **Ni** | **Pb** | **Cd** | **Al** | **Ba** |
| **Sealing** ^c^  **(N=8)** | Mean±SD | 20.35±42.23 | 4.23±2.52 | 17.32±30.87 | 0.41±0.35 | 0.02±0.01 | 121.82±142.50 | 0.76±0.40 |
|  | GM(GSD) | 7.65(3.55) | 3.67(1.75) | 5.73(4.62) | 0.33(1.90) | 0.02(1.42) | 87.66(2.10) | 0.63(2.10) |
|  | Range (Median) | 2.77 - 124.77 (5.30) | 1.86 - 9.39 (3.55) | 0.70 - 90.93 (4.53) | 0.17 - 1.22 (0.31) | 0.01 - 0.04 (0.02) | 43.84 - 470.66 (77.88) | 0.15 - 1.28 (0.80) |

^c^ Significant difference (p<0.05) of post-treatment process(ANOVA test)

**Abbreviation**: SD=Standard Deviation, GM=Geometric Mean, GSD=Geometric Standard Deviation, Cr=Chromium(Ⅵ), Zn=Zinc, Ni=Nickel, Pb=Lead, Cd=Cadmium, Al=Aluminum, Ba=Barium

**Table S12.** Heavy metals in post treatment-polishing process

| **Process**  **(N=14)** |  | **heavy metals (μg/㎥)** | | | | | | |
| --- | --- | --- | --- | --- | --- | --- | --- | --- |
|  |  | **Cr** | **Zn** | **Ni** | **Pb** | **Cd** | **Al** | **Ba** |
| **Polishing** ^c^  **(N=7)** | Mean±SD | 479.81±833.01 | 5.47±3.22 | 24.76±54.81 | 0.70±0.59 | 0.04±0.03 | 97.97±60.33 | 0.83±0.67 |
|  | GM(GSD) | 169.37(5.63) | 4.57(1.98) | 4.98(5.52) | 0.51(2.53) | 0.03(1.84) | 83.59(1.86) | 0.63(2.23) |
|  | Range (Median) | 6.62 – 2,358.41 (183.74) | 1.66 - 10.54 (5.66) | 0.87 - 148.71 (2.50) | 0.11 - 1.85 (0.60) | 0.01 - 0.09 (0.03) | 28.88 - 217.13 (75.93) | 0.20 - 1.90 (0.50) |
| **Non polishing** ^c^  **(N=7)** | Mean±SD | 5.71±1.74 | 11.05±4.37 | 5.89±2.32 | 0.44±0.33 | 0.03±0.01 | 91.52±23.16 | 0.67±0.42 |
|  | GM(GSD) | 5.50(1.34) | 10.35(1.47) | 5.54(1.46) | 0.34(2.17) | 0.03(1.45) | 89.00(1.29) | 0.56(2.03) |
|  | Range (Median) | 3.56 - 8.75 (5.19) | 6.46 - 18.09 (9.14) | 3.35 - 10.18 (5.13) | 0.12 - 1.07 (0.40) | 0.01 - 0.04 (0.03) | 65.77 - 119.09 (89.32) | 0.19 - 1.39 (0.64) |

^c^ Significant difference (p<0.05) of post-treatment process(ANOVA test)

**Abbreviation**: SD=Standard Deviation, GM=Geometric Mean, GSD=Geometric Standard Deviation, Cr=Chromium(Ⅵ), Zn=Zinc, Ni=Nickel, Pb=Lead, Cd=Cadmium, Al=Aluminum, Ba=Barium

**Table S13.** Heavy metals in post treatment-packaging & chromium cleaning process

| **Process**  **(N=16)** |  | **heavy metals (μg/㎥)** | | | | | | |
| --- | --- | --- | --- | --- | --- | --- | --- | --- |
|  |  | **Cr** | **Zn** | **Ni** | **Pb** | **Cd** | **Al** | **Ba** |
| **Drying & Packaging** ^c^  **(N=8)** | Mean±SD | 3.48±1.59 | 3.83±2.64 | 17.02±42.59 | 0.29±0.32 | 0.04±0.04 | 24.75±20.25 | 0.62±0.77 |
|  | GM(GSD) | 1.39(3.25) | 2.36(4.34) | 1.16(26.91) | 0.11(8.30) | 0.02(3.70) | 6.11(37.47) | 0.21(10.57) |
|  | Range (Median) | <LOD - 5.15 (3.72) | 0.08 - 8.45 (3.49) | <LOD - 122.31 (1.72) | <LOD - 0.93 (0.18) | <LOD - 0.13 (0.03) | <LOD - 54.03 (24.47) | <LOD - 2.41 (0.38) |
| **Chromium cleaning** ^c^  **(N=8)** | Mean±SD | 4.53±1.46 | 5.12±4.04 | 3.31±2.62 | 0.36±0.25 | 0.03±0.01 | 29.01±23.43 | 0.55±0.46 |
|  | GM(GSD) | 4.34(1.37) | 3.97(2.23) | 2.56(2.13) | 0.23(3.44) | 0.02(1.64) | 20.24(2.60) | 0.40(2.40) |
|  | Range (Median) | 2.64 - 7.31 (4.13) | 1.52 - 13.47 (4.50) | 1.05 - 7.39 (2.24) | 0.02 - 0.72 (0.40) | 0.01 - 0.05 (0.02) | 5.39 - 65.01 (21.18) | 0.11 - 1.56 (0.47) |

^c^ Significant difference (p<0.05) of post-treatment process(ANOVA test)

**Abbreviation**: SD=Standard Deviation, GM=Geometric Mean, GSD=Geometric Standard Deviation, Cr=Chromium(Ⅵ), Zn=Zinc, Ni=Nickel, Pb=Lead, Cd=Cadmium, Al=Aluminum, Ba=Barium

**Table S14.** Hood ventilation flow rate in pre-treatment & surface treatment process

| **Process** | **Flow rate (m^3^/s)** | | | | | | |
| --- | --- | --- | --- | --- | --- | --- | --- |
|  | **1 slot**  **(mean)** | **2 slot**  **(mean)** | **3 slot**  **(mean)** | **4 slot**  **(mean)** | **5 slot**  **(mean)** | **Total slot**  **(mean)** | **30 cm Distance**  **(mean)** |
| **Degreasing** | 1.88 - 2.34 (2.06) | 0.92 - 1.42 (1.21) | 0.32 - 2.55 (1.83) | 1.15 - 2.37 (1.87) | - | 0.32 - 2.55 (1.74) | 0.10 - 0.16 (0.12) |
| **Etching & Neutralization** | 0.79 - 1.34 (0.92) | 1.78 - 2.46 (2.29) | 1.55 - 2.43 (1.82) | 1.38 - 2.67 (2.03) | - | 0.79 - 2.67 (1.77) | 0.11 - 0.17 (0.14) |
| **Soft Anodizing_1^*^** | 0.63 - 1.57 (1.046) | 1.52 - 2.13 (1.73) | 1.37 - 2.44 (2.05) | 2.01 - 2.52 (2.22) | - | 0.63 - 2.52 (1.76) | 0.10 - 0.19 (0.14) |
| **Soft Anodizing_2^**^** | 0.38 - 1.72 (1.17) | 1.76 - 1.99 (1.85) | 3.09 - 3.99 (3.62) | 0.99 - 2.57 (1.90) | - | 0.38 - 3.99 (2.14) | 0.08 - 0.18 (0.13) |
| **Hard Anodizing_1^*^** | 1.01 - 1.93 (1.59) | 1.57 - 2.70 (2.05) | 2.34 - 2.69 (2.53) | 1.42 - 2.85 (2.30) | - | 1.01 - 2.85 (2.12) | 0.04 - 0.18 (0.10) |
| **Hard Anodizing_2^**^** | 1.42 - 1.92 (1.645) | 2.16 - 2.78 (2.54) | 1.35 - 3.12 (2.44) | 1.69 - 3.13 (2.70) | - | 1.35 - 3.13 (2.33) | 0.12 - 0.22 (0.17) |
| **Chromium plating** | 1.67 - 2.79 (2.07) | 1.01 - 1.44 (1.21) | 3.78 - 4.98 (4.43) | 2.05 - 3.69 (2.94) | - | 1.01 - 4.98 (2.66) | 0.13 - 0.22 (0.17) |

^*^ 1= Medium size of bath

^**^ 2=Large size of bath

dotted line: group of process

**Table S15.** Hood ventilation flow rate in post-treatment process

| **Process** | **Flow rate (m^3^/s)** | | | | | | |
| --- | --- | --- | --- | --- | --- | --- | --- |
|  | **1 slot**  **(mean)** | **2 slot**  **(mean)** | **3 slot**  **(mean)** | **4 slot**  **(mean)** | **5 slot**  **(mean)** | **Total slot**  **(mean)** | **30 cm Distance**  **(mean)** |
| **Coloring_1^*^** | 1.07 - 1.58 (1.30) | 0.63 - 1.06 (0.88) | 1.17 - 2.23 (1.61) | 0.94 - 1.35 (1.09) | - | 0.63 - 2.23 (1.22) | 0.10 - 0.17 (0.12) |
| **Coloring_2^**^** | 0.86 - 1.39 (1.07) | 0.84 - 1.34 (1.03) | 1.89- 2.13 (1.98) | 0.96 - 1.53 (1.16) | - | 0.84 - 2.13 (1.31) | 0.08 - 0.13 (0.12) |
| **Sealing** | 1.05 - 1.53 (1.30) | 1.04 - 1.24 (1.11) | 2.06 - 2.69 (2.28) | 1.02 - 1.56 (1.20) | - | 1.02 - 2.69 (1.47) | 0.09 - 0.18 (0.14) |
| **Polishing** | 0.67 - 1.32 (0.92) | 0.90 - 1.57 (1.24) | 1.94 - 2.72 (2.32) | 1.37 - 2.66 (2.28) | 1.68 - 2.25 (1.87) | 0.67 - 2.72 (1.69) | 0.10 - 0.16 (0.14) |
| **Non polishing** | 0.80 - 1.14 (0.96) | 0.35 - 1.09 (0.75) | 1.52 - 2.57 (2.08) | 0.70 - 1.68 (1.02) | - | 0.35 - 2.57 (1.20) | 0.12 - 0.18 (0.15) |
| **Chromium cleaning** | 0.93 - 2.92 (2.11) | 1.15 - 1.48 (1.28) | 2.73 - 3.72 (3.34) | 1.53 - 2.86 (2.25) | - | 0.93 - 3.72 (2.24) | 0.11 - 0.25 (0.18) |

^*^ 1= Medium size of bath

^**^ 2=Large size of bath


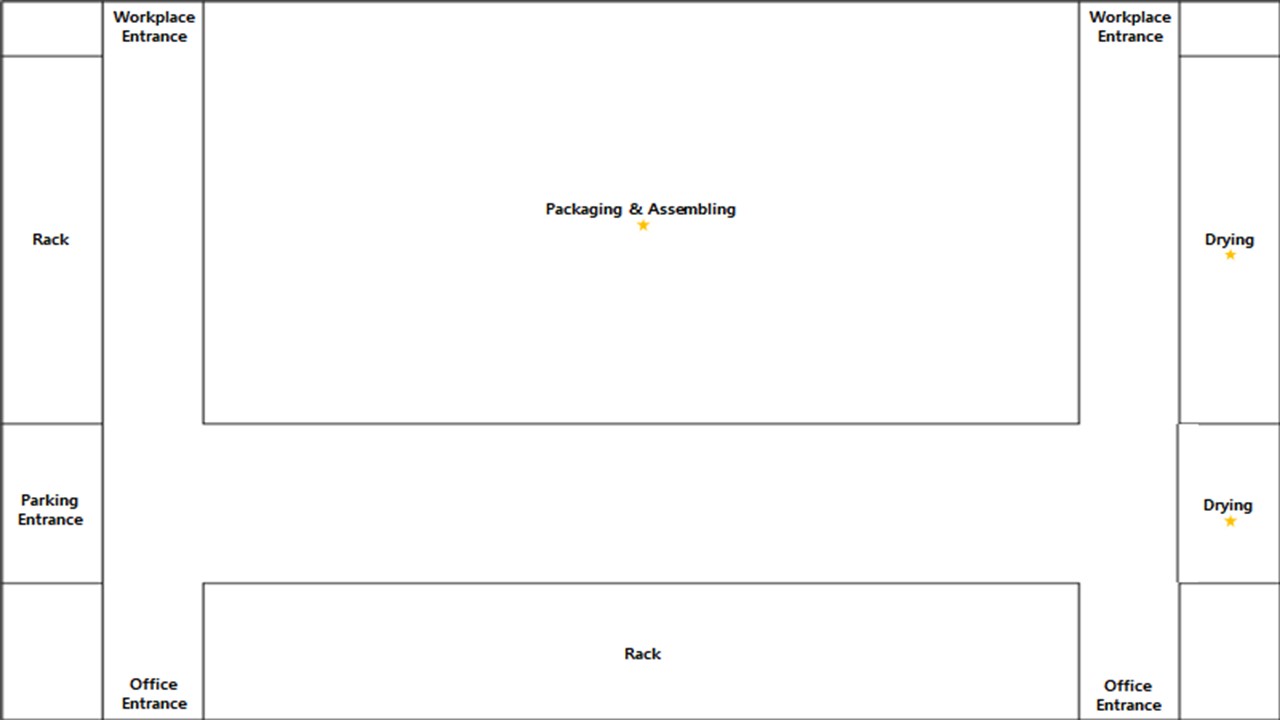


**Fig. S1.** Anodizing and electroplating schematic and sample location 2: the icons are the sampling locations (orange star: other processes).

**Note**: Workplace(31m×14m)


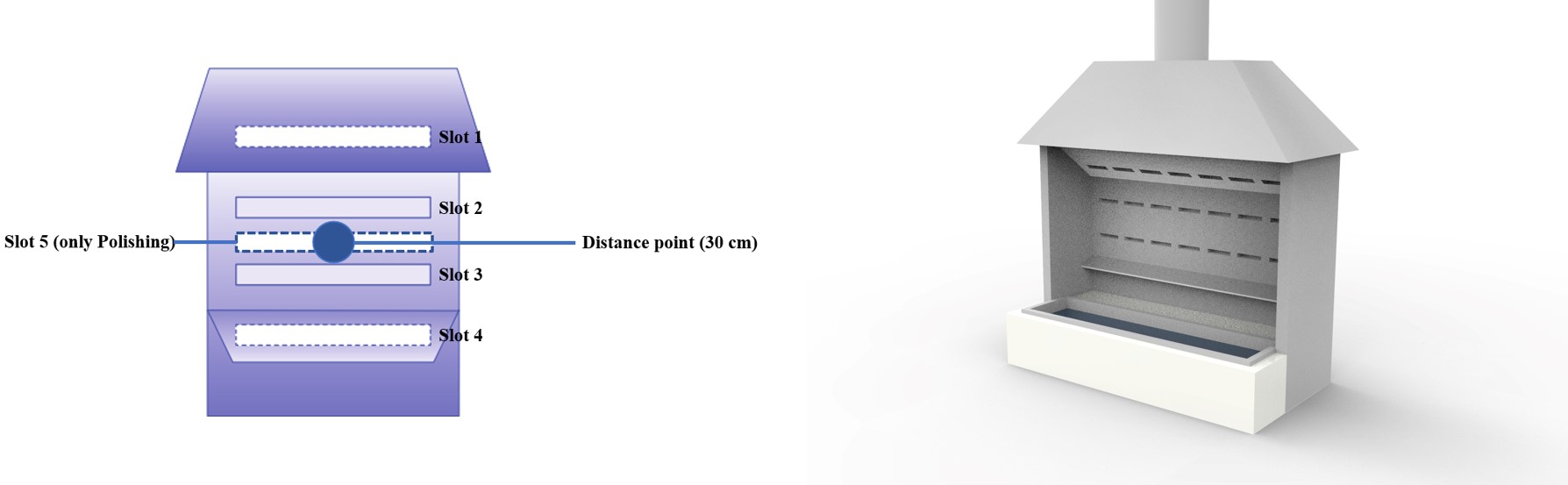


**Fig. S2.** Schematic diagram of the hood and bath.
